# The transcriptome of *Utricularia vulgaris*, a rootless plant with minimalist genome, reveals extreme alternative splicing and only moderate sequence similarity with *Utricularia gibba*

**DOI:** 10.1186/s12870-015-0467-8

**Published:** 2015-03-07

**Authors:** Jiří Bárta, James D Stone, Jiří Pech, Dagmara Sirová, Lubomír Adamec, Matthew A Campbell, Helena Štorchová

**Affiliations:** Faculty of Science, University of South Bohemia, Branišovská 31, České Budějovice, 37005 Czech Republic; Institute of Experimental Botany CAS, Rozvojová 263 6- Lysolaje, Praha, 16502 Czech Republic; Institute of Botany CAS, Zámek 1, Průhonice, 25243 Czech Republic; Institute of Botany CAS, Section of Plant Ecology, Dukelská 135, Treboň, 37982 Czech Republic; Institute of Fundamental Sciences, Massey University, Private Bag 11222, Palmerston North, 4442 New Zealand

**Keywords:** Transcriptome, Root-associated genes, Alternative splicing, *Utricularia vulgaris*

## Abstract

**Background:**

The species of *Utricularia* attract attention not only owing to their carnivorous lifestyle, but also due to an elevated substitution rate and a dynamic evolution of genome size leading to its dramatic reduction. To better understand the evolutionary dynamics of genome size and content as well as the great physiological plasticity in this mostly aquatic carnivorous genus, we analyzed the transcriptome of *Utricularia vulgaris*, a temperate species with well characterized physiology and ecology. We compared its transcriptome, namely gene content and overall transcript profile, with a previously described transcriptome of *Utricularia gibba*, a congener possessing one of the smallest angiosperm genomes.

**Results:**

We sequenced a normalized cDNA library prepared from total RNA extracted from shoots of *U. vulgaris* including leaves and traps, cultivated under sterile or outdoor conditions. 454 pyrosequencing resulted in more than 1,400,000 reads which were assembled into 41,407 isotigs in 19,522 isogroups. We observed high transcript variation in several isogroups explained by multiple loci and/or alternative splicing. The comparison of *U. vulgaris* and *U. gibba* transcriptomes revealed a similar distribution of GO categories among expressed genes, despite the differences in transcriptome preparation. We also found a strong correspondence in the presence or absence of root-associated genes between the *U. vulgaris* transcriptome and *U. gibba* genome, which indicated that the loss of some root-specific genes had occurred before the divergence of the two rootless species.

**Conclusions:**

The species-rich genus *Utricularia* offers a unique opportunity to study adaptations related to the environment and carnivorous habit and also evolutionary processes responsible for considerable genome reduction. We show that a transcriptome may approximate the genome for gene content or gene duplication estimation. Our study is the first comparison of two global sequence data sets in *Utricularia*.

**Electronic supplementary material:**

The online version of this article (doi:10.1186/s12870-015-0467-8) contains supplementary material, which is available to authorized users.

## Background

Members of the rootless genus *Utricularia* (Lentibulariaceae) are the most versatile and cosmopolitan among carnivorous plants, exhibiting great morphological and ecophysiological plasticity [1-3]. Approximately 50 species of *Utricularia* are aquatic or amphibious, growing in standing, nutrient-poor humic waters. While their ecology and carnivorous habit have been researched previously [3], increasing attention has been given to the peculiarities of *Utricularia* genomes - miniature size in many species within the family [4,5], highly increased nucleotide substitution rates across the genomes of all three cellular compartments: mitochondrial, plastid, and nuclear [6-9], and to the extremely dynamic evolution of genome size at the level of species or even single populations [4,10]. *Utricularia gibba* possesses one of the smallest haploid angiosperm genomes known, approximately one-half that of *Arabidopsis thaliana*, with chromosomes of bacterial size [4,5]. *U. gibba* was the subject of the first broad survey of nuclear gene transcripts in *Utricularia* species [11,12], revealing several interesting aspects of their physiology and morphology. Supporting physiological data, the global transcript analysis revealed specific expression patterns of genes involved in respiration, DNA repair, ROS detoxification, and nutrient uptake in different plant tissues. The sequencing and analysis of the *U. gibba* genome [13] additionally revealed a compressed genome architecture with highly reduced intergenic regions and nearly free of retrotransposons.

*Utricularia* spp*.* have since been identified as prime candidates for further research on the complexities of plant ecophysiology associated with carnivory, metagenomic surveys of trap microbial communities, novel plant nitrogen/nutrient utilization pathways, the ecology of prey attraction, whole-plant and trap comparative development, and the evolution of a minimalist angiosperm genome [3,5,14-20]. *Utricularia gibba*, however, is not a good candidate species for many ecological and physiological experiments due to its minute size and extremely small traps. We have therefore chosen the ecologically well-characterized temperate *Utricularia vulgaris* [3,16-18] as our model for a broad transcriptome analysis. Its ecophysiology is subtly but meaningfully distinct from that of *U. gibba*, offering the possibility for a comprehensive comparison of genome-wide expression patterns between the two species.

In this study, we report the results of 454 GS-FLX Titanium sequencing of a polyA-selected and normalized cDNA library from *U. vulgaris*, derived from a pooled sample of multiple tissue types, including functional annotation of expressed gene content. We compared this transcriptome to the *U. gibba* transcriptome [12] and showed that, despite different methods of preparation and tissue composition, the overall gene expression pattern and gene distribution among GO categories were very similar between the two species. We also analysed several cases of alternative splicing (AS) in the *U. vulgaris* transcriptome, including a gene for which this post-transcriptional process has not been investigated in any plant species.

Although any transcriptome should be viewed as incomplete, it may serve as an acceptable proxy for the genome in a species without complete genomic information, such as *U. vulgaris*, provided that it is prepared from multiple tissues and various environmental conditions [21,22]. We demonstrate the usefulness of the *U. vulgaris* transcriptome for the identification of gene losses and duplications during the course of evolution of the genus *Utricularia*.

## Results

### Transcriptome assembly

In total, 1,405,703 reads were generated by 454 pyrosequencing of the *U. vulgaris* normalized cDNA library, 1,389,835 of them passed built-in quality filtering. 91.5% of the initial, raw reads were assembled by Newbler 2.7 and produced 19,522 isogroups containing 41,407 isotigs, roughly corresponding to the individual transcripts. In addition, 64,188 singletons longer than 100 nt were obtained. Isotigs and singletons were combined together into a unique transcript (UT_U.vulgaris) data set representing the *U. vulgaris* transcriptome. To facilitate the comparison between our data and the *U. gibba* transcriptome published by [12], raw reads of *U. gibba* were downloaded from DNA Data Bank of Japan (DDBJ) under the submission SRA029151 and assembled by Newbler 2.7 using the same parameters as adopted for the *U. vulgaris* transcriptome (UT_U.gibba). Table 1 compares the transcriptome assemblies of the two species. Our *U. vulgaris* data set consisted of nearly twice as many raw reads, a higher proportion of which assembled into contigs, than the *U. gibba* dataset. The *U. vulgaris* assembly also resulted in a higher number of isogroups and much higher (about three fold) number of isotigs. Furthermore, our *U. vulgaris* assembly produced only 64,188 singletons compared to the 99,900 singletons remaining after *de novo U. gibba* assembly. The *U. vulgaris* data set contained about 2.1 isotigs per isogroup, whereas only 1.2 isotigs per isogroup, on average, were found in the *U. gibba* assembly. The much higher number of isotigs in the *U. vulgaris* transcriptome, both relative (per isogroup) and absolute, was at least partly caused by the method of cDNA library preparation. Our *U. vulgaris* cDNA library was normalized, increasing the number of rare transcripts represented by isotigs.

**Table 1:** **Transcriptomes comparison**

	***Utricularia vulgaris***	***Utricularia gibba***
Biological source of RNA	Shoots, cultivated under sterile conditions	Shoots and flowers natural conditions
cDNA library preparation	Oligo dT enrichment normalized library	Oligo dT enrichment without normalization
inputFile NumReads	1 405 703	817 792
numAlignedReads	1 271 732; 91.5% of total	707 880; 86.56% of total
Number of Assembled	1 151 110	638 520
Number of Partially assembled	120 448	69 288
Number of Singletons	64 188	99 900
Number Outlier (e.g. chimeras)	23 169	9967
Number of TooShort Reads	30 086	6
Number of Isogroups	19 522	13 591
Avg Contig Count	2.6	1.4
Number With One Contig	11 168; 57.2%	11 676; 85.9%
Avg Isotig Count	2.1	1.2
Number With One Isotig	11 213	11 705
Number of Isotigs	41 407	16 465
Avg Contig Count	3.4	1.6
Largest Contig Count	18	13
Number With One Contig	11 168	11 676
Avg Isotig Size	1 514	705
N50 Isotig Size	1905	839
Largest Isotig Size	12 895	3 326
Number Of Large Contigs	22 001	7 763
Avg Large Contig Size	962	819
Number Of all Contigs	51 270	19 120

*U. vulgaris* and *U. gibba* transcriptomes assembled by Newbler 2.7.

### Transcriptome annotation

39,006 *U. vulgaris* isotigs (96%) gave significant BLAST hit against the NCBI nr protein database (BLASTX algorithm, e-value cutoff 10^−5^). These sequences were further annotated using the BLAST2GO annotation pipeline. 30,392 isotigs (73% of total isotigs) were successfully annotated. 9,794 isotigs (33% of annotated isotigs) were assigned with enzyme codes (E.C.). The average level of annotations in GO hierarchy was 5,868. The total number of assigned Gene Ontology terms was 212,122 (Table 2).

**Table 2:** **GO Annotation summary**

	**Isotigs**		**Singletons**	
	***U.vulgaris***	***U.gibba***	***U.vulgaris***	***U.gibba***
NCBI nr	96%	89%	40%	47%
Number of GO terms	212 122	95 741	90 735	264 218
GO annotation	73%	75%	25%	39%
Biological process	54%	52%	54%	53%
Molecular function	13%	12%	14%	13%
Cellular component	34%	36%	32%	34%
E.C. assignment	33%	28%	25%	33%

Values in % indicate the percentage of sequences⁄groups with one or more significant blast hits/ annotations based on an e-value cut-off of 10^−5^.

Of the total 58,363 *U. vulgaris* singletons, 23,212 (40%) gave a significant BLAST hit against the NCBI nr protein database under the same parameters as used for isotigs. 14,536 singletons (25% of total singletons) were successfully annotated and 4,121 singletons (28% of annotated singletons) were assigned with E.C. The average level of annotations in GO hierarchy was 5,791. The total number of assigned Gene Ontology terms was 90,735. The much lower proportion of *U. vulgaris* singletons yielding significant BLAST hits, compared with the isotigs, may be due to their short sizes and also due to the presence of transcripts derived from microbes without any NCBI record.

The results of the GO annotations of the UT_U.gibba transcriptome are given in Table 2. The proportion of annotated isotigs is a bit lower and the proportion of annotated singletons is a bit higher in *U. gibba* than in *U. vulgaris*. This difference results from a higher amount of unassembled reads in UT_U.gibba relative to UT_U.vulgaris. The proportion of isotigs with an assigned E.C. was also higher in *U. vulgaris* than in *U. gibba*.

**Figure 1:**
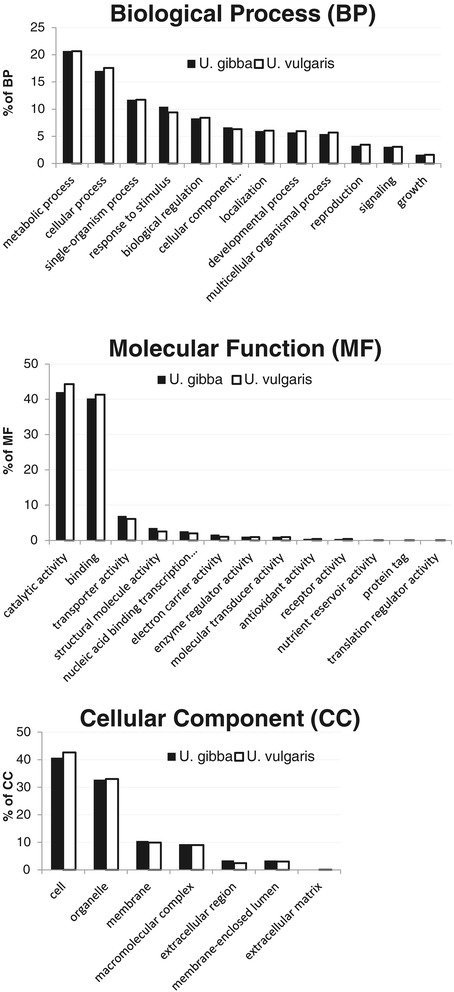
**Distribution of GO categories.** The comparison of the distribution of unique transcripts (isotigs and singletons) between *U. gibba* and *U. vulgaris* transcriptomes in three main GO categories.

Despite of the differences in cultivation conditions, plant tissues used for RNA extraction, cDNA library preparation and assembly parameters, the general partition of isotigs into basic KEGG categories was very similar between the two *Utricularia* species (Figure 1). “Catalytic activity” and “Binding” were the prevalent categories among Molecular Function. “Cell” and “Organelle” dominated in the Cellular Component section. Abundant categories “Metabolic Process” and “Cellular” were followed by slightly less numerous categories “Response to stimulus” and “Biological regulation”. The high representation of the “Single-organism process” category appeared due to co-existing microbes.

We summarized the results of our *U. vulgaris* transcriptome assembly and annotation and created a web-accessible database (http://utricularia.prf.jcu.cz/index.php) which can be easily searched by BLAST or annotation.

### The composition of transcriptomes

**Figure 2:**
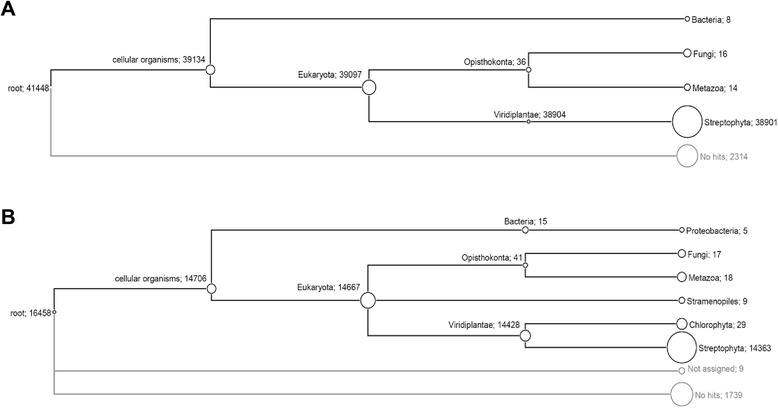
**Taxonomic assignment.** Dendrogram showing number of MEGAN assigned *U. vulgaris* **(A)** and *U. gibba* **(B)** isotigs.

More than 99% of the isotigs with significant hits were assigned by MEGAN to plants (Streptophytes) in both *U. vulgaris* and *U. gibba*. All remaining isotigs (38 in *U. vulgaris* and 87 in *U. gibba*) belonged to Fungi, Metazoa, unicellular eukaryotes, and prokaryotes (Figure 2). The taxonomic diversity of singletons was much higher: 5.3% and 10.6% of singletons with significant hits were assigned outside the Streptophytes in *U. vulgaris*, and *U. gibba*, respectively (Additional file 1). The non-plant sequences were mostly derived from microbial commensals, as well as a minor fraction from animal (fish, worm) RNA contamination. The very low proportion of prokaryotic sequences was due to the polyA+ RNA used to prepare cDNA. As prokaryotic mRNAs rarely contain polyA+ tails, they were mostly eliminated. The proportion of non-plant transcripts is probably higher among singletons, because many of them may not have produced statistically significant hits due to incomplete microbial records in public databases. The abundance of microbe-derived transcripts was higher in the *U. gibba* transcriptome prepared only from plants grown under natural conditions and colonized with microbes. In contrast, the *U. vulgaris* transcriptome was constructed from a pooled RNA sample prepared from the plants cultivated under both sterile and non-sterile conditions.

### Large isogroups and alternative splicing

The isogroups containing numerous isotigs may include transcripts derived from several or many loci, e.g. retroposons, or from transcripts undergoing AS [23]. The *U. vulgaris* transcriptome contained six isogroups with > 100 isotigs, 23 isogroups with > 45 isotigs, and 332 isogroups with > 10 isotigs. The largest, isogroup 00018 in *U.vulgaris*, included 480 isotigs derived from various members of a large *BETA GLUCOSIDASE* gene family.

In contrast, the *U. gibba* assembly contained zero isogroups with > 100 isotigs, only two isogroups with > 45 isotigs, and 17 isogroups with > 10 isotigs (Additional file 2). The main reason for such a high difference in the number of large isogroups with many isotigs between the UT_U.vulgaris and UT_U.gibba transcriptome assemblies seems to be the method of cDNA preparation. Normalization of the cDNA library led to the enrichment of rare transcripts in *U. vulgaris*, including alternatively spliced mRNAs. The largest isogroup in the UT_U.gibba assembly, which was generated without a cDNA normalization step, contained only 68 isotigs, representing transcripts coding for the small subunit of Rubisco, the most abundant protein on Earth. When read counts are extremely high, as in the case of Rubisco, sequencing errors occur in multiple reads which are then assembled into separate, artifactual contigs. Some large isogroups in *U. gibba* also represented transcripts derived from multiple loci-e.g. isogroup 00005 (*KETOACYL COA SYNTHASE* family) [24] or the isogroups 00002 and 00012, which gave no hits in BLAST searches of NCBI databases, but yielded multiple hits against the *U. gibba* genome draft (CoGe-id36222).

Alternative splicing appears to be the main reason for the transcript abundance and diversity in many of the largest isogroups in *U. vulgaris* and in *U. gibba*. These isotigs contain contigs corresponding to *Arabidopsis* exons and also numerous contigs which may be assigned to introns based on their position between two exons. Additional file 2 compares the 23 and 17 largest isogroups of *U. vulgaris* and *U. gibba*, respectively. They represent various genes or gene families belonging to similar structure and function categories.

Only one large isogroup (00008) appears to be the same in both *Utricularia* species. It encodes a family of ATP dependent RNA helicases. Its *Arabidopsis* homologs (At5g11170, At5g11200) are involved in a wide range of RNA metabolism including pre-mRNA splicing, mRNA transport, turnover, translation initiation etc. [25,26]. They undergo AS, as documented by genome-wide analysis of transcript variants [27]. Five contigs of the isogroup 00008 in *U. vulgaris* match *Arabidopsis* exons, suggesting extensive AS of transcripts derived from at least two related genes. The more than four fold higher isotig count of the 00008 isogroup in *U. vulgaris* than in *U. gibba* may again reflect a significant enrichment in rare transcripts due to cDNA normalization of *U. vulgaris* transcriptome, or reflect a lower extent of AS in *U. gibba*. Three other isogroups of *U. vulgaris* could participate in the control of AS, including the isogroup 00013, encoding a homolog of *AFC2* protein kinase, which underwent extreme AS (producing multiple splice variants from the same primary transcript) in *Arabidopsis* [27]. The remaining large isogroups with AS code for membrane proteins with multiple domains, proteins involved in protein degradation, or fulfilling regulatory functions. Two large isogroups (00020, 00061) were assigned to retroposons in the *U. vulgaris* transcriptome. No large isogroup corresponding to transposons or retroposons was found in the *U. gibba* transcriptome, however three single isotigs were.

### Two isogroups with extreme alternative splicing

**Figure 3:**
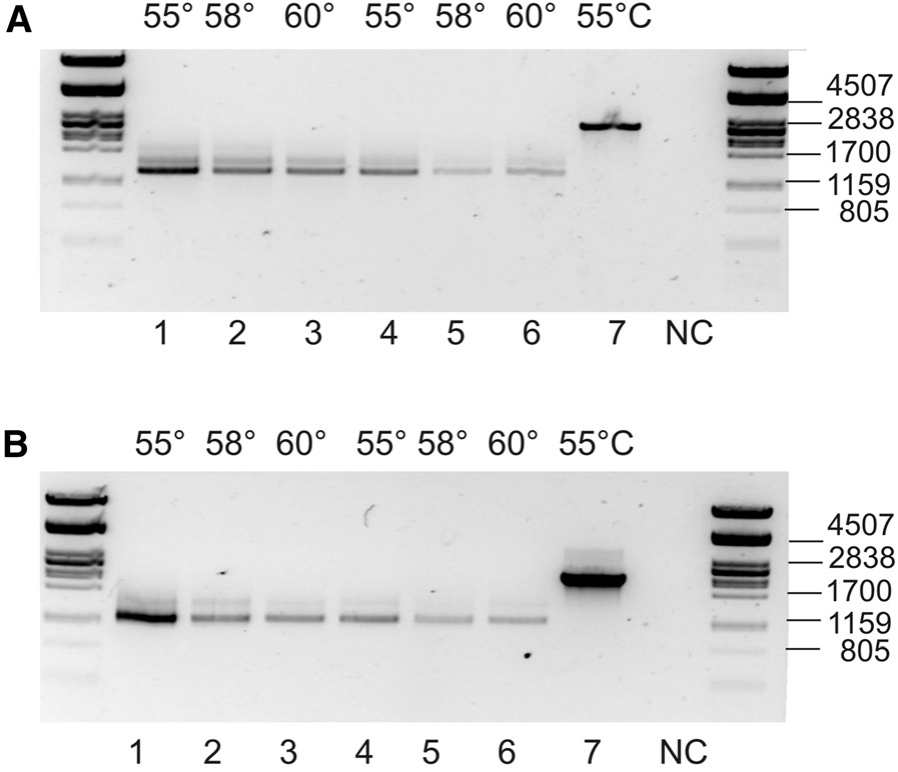
**PCR amplification with the** ***LCB-1-P lyase*** **specific primers.** An agarose gel (1.2%) electrophoresis of PCR fragments amplified from the gene encoding LCB-1-P lyase (isogroup 000007) in *U. vulgaris* with different templates. 1–6: cDNA prepared from RNA extracted from two different plant individuals, 7: genomic DNA. NC: negative control with water instead of DNA. **(A)** PCR with exon-specific primers UV405_F1 and UV405_R1. **(B)**. PCR with intron-specific primer UV405_F2 and exon-specific primer UV405_R1. Annealing temperature is indicated above the lanes. Standard of molecular weights is shown on the both sides of the gel.

We selected two isogroups of *U. vulgaris* with very high isotig counts for more detailed analysis. After aligning all 277 isotigs of the isogroup 00007, we found that all of them were derived from the same locus, because only one sequence variant (contig) corresponded to each exon of the homologous *Arabidopsis* gene, At1g27980, coding for sphingoid long-chain base 1-phosphate lyase (LCB-1-P lyase) (Additional file 3). We assigned eight contigs to eight introns based on a comparison with the homologous *Arabidopsis* gene. The retention of variable numbers of introns was responsible for the observed extreme AS in this isogroup. Only one isotig 00648 contained the correct ORF with genetic information for a functional protein. To confirm AS experimentally, we designed primers targeted to exon 6 or intron 6 (forward) and exon 15 (reverse) and ran PCR (Figure 3). The size of PCR fragment generated from genomic DNA (2.4 kb) with exon-specific primers UV405_F1 and UV405_R1 agreed with the expected size of this genomic region (2,353 bp). The amplification of cDNA produced a strong band (1.3 kb) corresponding to correctly spliced mRNA with no introns (1,377 bp) and several weak upper bands most likely derived from partially spliced mRNA with retained introns. The primers spanning from intron 6 to exon 15 (UV405_F2 and UV405_R1) produced a PCR fragment from genomic DNA as well as one strong band (1.1 kb) and a few weaker ones from cDNA. The strong band amplified from cDNA provided evidence for intron 6 retention, because no amplification with this primer pair could occur if only correctly spliced mRNA were present in the transcript pool.

**Figure 4:**
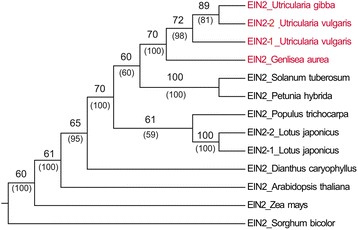
**Phylogenetic analyses of** ***EIN2*** **.** MP and ML tree constructed from the alignment of partial *EIN2* sequences across angiosperms exhibited the same topology. Bootstrap supports calculated from 1000 pseudoreplicates are shown above branches (MP) or below branches in parentheses (ML).

To achieve the correct assembly of alternative transcripts in a species without reference genome is very difficult. It becomes even more challenging if multiple similar paralogous genes are transcribed and alternatively spliced. In such cases, chimeric misassembled contigs are frequently generated [28]. The isogroup 00006 homologous to the *ETHYLENE INSENSITIVE 2 EIN2* gene (At5g03280) in *Arabidopsis* is an example of the mixture of alternatively spliced transcripts derived from at least two loci. We identified contigs corresponding to the exons and introns of the *EIN2* gene. Several putative exons existed in two sequence variants and occurred in chimeric isotigs. We confirmed the occurrence of two *EIN2* gene copies by direct sequencing of *U. vulgaris* genomic DNA. We designed two primer pairs UV304_F1, R1 and UV308_F1, R1 (Additional file 4) and amplified and sequenced a part of exon 7 from both *EIN2* paralogs. The alignment (1,360 bp) of *U. vulgaris* sequences with *EIN2* homologs across angiosperms was used in phylogenetic analysis to generate MP and ML trees (Figure 4). The trees constructed by both methods showed the same topology and confirmed a relatively recent duplication of *EIN2*, preceding the divergence of *U. vulgaris* and *U. gibba*. We found only one *EIN2* homolog in the *U. gibba* genome (CoGe-id36222).

The ratio of non-synonymous and synonymous substitutions (Ka/Ks) in the pairwise comparison between *EIN2-1* and *EIN2-2* of *U. vulgaris* was 0.41, between *EIN2* of *U. vulgaris* and *EIN2* of *U. gibba* was 0.44 (for both *U. vulgaris EIN2* paralogs). The data suggest no variation in evolutionary constraints.

### Putative orthologs between *U. vulgaris* and *U. gibba*

We performed a reciprocal BLAST hit search to identify putative orthologs between the UT_U.vulgaris transcriptome and a 19475-mRNA database derived from the genomic draft of *U. gibba*, which represents an *in silico* transcriptome of this species. We chose the *U. gibba* transcriptome derived from a genomic draft, because it supposedly represented more complete set of transcripts than the experimental transcriptome UT_U. gibba.

**Figure 5:**
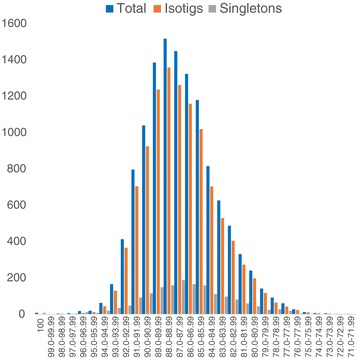
**Ortholog similarity distribution.** The distribution of putative orthologous pairs between *U. gibba* and *U. vulgaris* according to their sequence similarity. Each bar represents the number of pairs of a given similarity spanning 1% interval. Blue–all orthologous pairs, orange–pairs with *U. vulgaris* isotigs, grey–pairs with *U. vulgaris* singletons.

We identified 12,267 putative orthologous pairs, 10,600 of them contained *U. vulgaris* isotigs and the remaining 1,667 pairs contained *U. vulgaris* singletons. The orthologs represented about 42.9% of all genes annotated in the *U. gibba* genomic draft. We ordered putative orthologous pairs between *U. gibba* and *U. vulgaris* according to the sequence similarity of the regions aligned by BLAST. The distribution of orthologs assigned to individual similarity classes is shown in Figure 5. Most orthologous pairs exhibited a sequence similarity of 85%-90%, whether or not they included *U. vulgaris* isotigs or singletons. Singletons are much shorter than an average isotig (1,514 bp; Table 1), thus they often represent incomplete transcripts. Their sequence similarity depends on whether they are derived from a more or less conserved part of the gene, it does not reflect the similarity across an entire gene. For this reason, we performed the following analyses of the most conserved orthologs with the pairs containing only *U. vulgaris* isotigs, not singletons.

**Figure 6:**
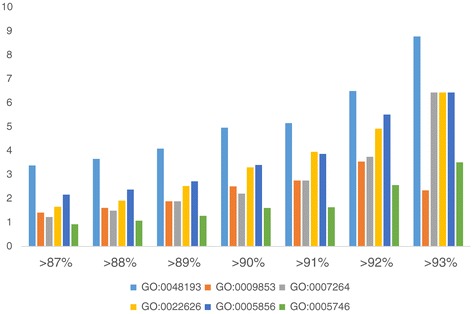
**GO enrichment.** The enrichment of particular GO categories (in % of total GO categories) in the subsets of orthologous pairs between *U. vulgaris* and *U. gibba* with ascending sequence similarity. GO:0048193 Golgi vesicle transport; GO:0009853 Photorespiration GO: 0007264 Small GTPase mediated signal transduction; GO: 00022626 Cytosolic ribosomes; GO:0005856 Cytoskeleton; GO: 0005746 Mitochondrial respiratory chain.

Because the overall sequence similarity of putative *U. vulgaris*-*U. gibba* orthologs was rather low (median 87%), we investigated which GO categories were enriched among the most conserved orthologous pairs. GO enrichment (AgriGo) [29] analysis of the most conserved orthologs (with similarity higher than 93%) against all orthologs identified 36 significantly enriched GO categories (Additional file 5). They belonged to the genes encoding proteins conserved across all angiosperms (ribosomal proteins, tubulins, small GTP-binding proteins, mitochondrial respiratory chain proteins, etc.). Their proportion in respective similarity classes of putative orthologs increased with increasing sequence similarity (Figure 6). Detailed inspection of GO categories enriched among highly conserved orthologs between *U. gibba* and *U. vulgaris* revealed genes which were less similar to their *Arabidopsis* counterparts than the rest of the highly conserved orthologs, namely *MYOSIN XI B* (homolog of At1g04160) and *TIP GROWTH DEFECTIVE 1 (TIP1*) (a homolog of At5g20350). Interestingly, both genes play a role in root hair development in *Arabidopsis*. As neither *U. gibba* nor *U. vulgaris* produce roots, it is probable that the two genes have gained a novel or modified functions in *Utricularia*, explaining why their sequences are highly similar between both *Utricularia* species, but less similar *to Arabidopsis* homologs.

### Root-specific genes in rootless *Utricularia*

As *Utricularia vulgaris* does not form roots, some of the genes involved in root development and function might have been lost. Ibarra-Laclette et al. [13] published a list of the genes associated with root in *A. thaliana*, but not found in the genome of *U. gibba*. They include *MYB* transcription factors, MADS box genes, cell-wall-associated kinases, nitrate transporter etc. (Ibarra Laclette et al. [13]). We selected the root-associated genes absent in *U. gibba,* supplemented additional genes exclusively or predominantly expressed in the roots of *A. thaliana* and checked their presence among putative orthologs identified by BLASTX-TBLASTN reciprocal BLAST search between *A. thaliana* protein data set (TAIR10_prot_20101214) and the UT_U.vulgaris transcriptome. We found a strong correspondence between the absence of particular genes in the *U. gibba* genome [13] and their absence in the *U. vulgaris* transcriptome (Additional file 6). Moreover, we did not find the counterparts of additional *Arabidopsis* root-associated genes in the *U. vulgaris* transcriptome, notably transcription factors involved in root hair (e.g. *ROOT HAIR DEFECTIVE 6*-*RHD6*, *WEREWOLF*–*WER*) or root cap development (e.g. *BEARSKIN 1–BRN1*, *FEZ*, *SOMBRERO*–*SMB*). These genes were also missing in the *U. gibba* genome (CoGe-id36222). On the other hand, some genes such as *AUXIN RESPONSE FACTOR 4* (*ARF4*) or *INDOLE-3-ACETIC ACID INDUCIBLE* (*IAA*) genes found putative orthologs in both the *U. vulgaris* transcriptome and the *U. gibba* genome (Additional file 6). Six of 13 *U. vulgaris* root-associated genes (isotigs or single reads) under study contained complete ORFs, the rest of isotigs represented partial sequences, which reflected an overall incompleteness of experimental transcriptomes.

Because the transcriptomes are incomplete, the absence of an ortholog in the transcriptome, by itself, is not the evidence of its absence in the corresponding genome. Thus, we may not exclude the possibility that the absence of any respective gene in the *U. vulgaris* transcriptome was caused by its low or missing transcription. However, the coincident absence of 48 root-associated genes and concordant presence of 11 root-associated genes in the *U. vulgaris* transcriptome and the *U. gibba* genome suggest that, at least in the case of root genes, the UT_U.vulgaris transcriptome reflects the gene content of the *U. vulgaris* genome.

Interestingly, two copies of the gene *AUXIN RESPONSE FACTOR 16* (*ARF16*), duplicated in the *U. gibba* genome [13], were also found in the *U. vulgaris* transcriptome. The full agreement between the sets of root-associated genes lost in *U. gibba* and *U. vulgaris* and the concordance of the genes duplicated in both species support the notion that deletions and duplications of genes involved in root-associated genes occurred before the divergence of the two *Utricularia* species.

## Discussion

### Transcriptome comparison

Recent progress in next generation sequencing makes it possible to sequence the genomes and transcriptomes of non-model plants to an unprecedented extent. The 1000 plants (one KP or 1KP) initiative (https://sites.google.com/a/ualberta.ca/onekp) is just one example of current efforts. The genomic draft of *U. gibba* [13] has attracted attention because it represents one of the smallest genomes in the plant kingdom and opened the possibility to study the mechanisms responsible for genome contraction in plants. The availability of a sequenced genome and an experimental transcriptome of *U. gibba* generated by 454 pyrosequencing from various organs [12], made *U. gibba* a suitable species for comparative transcriptomics in *Utricularia*. We chose a temperate congener *U. vulgaris* as a counterpart for comparison. Both species share an aquatic carnivorous life style, lack of roots, and display rapid apical shoot growth, but exhibit partly distinct ecophysiology (turion formation in *U. vulgaris*, possible terrestrial life in *U. gibba*; see [1].

We utilized the two kinds of transcriptomes available for *U. gibba* in our comparative studies. The *in silico* transcriptome derived from the genomic draft is assumed to be more complete than the experimental transcriptome, which is known to lack transcripts due to low or missing gene expression [21]. However, an *in silico* transcriptome is only as good and complete as the annotation of the genome of interest. It may also erroneously assign virtual transcript to a pseudogene which is not expressed. In contrast, an experimental transcriptome is comprised of real transcripts able to capture alternatively spliced transcripts derived from the same gene. Considering advantages and disadvantages of the two kinds of transcriptomes, we decided to prefer the *in silico* transcriptome for the identification of putative orthologs between *U. gibba* and *U. vulgaris*. In contrast, experimental transcriptome of *U. gibba* [12] was used to compare expressed gene categories and also to identify alternatively spliced transcripts.

The transcriptome of *U. gibba* [12] was prepared from inflorescences in addition to submersed parts of plants, but, unlike *U. vulgaris* transcriptome, it did not include sterile plants. Another distinction was the application of cDNA normalization to the construction of the *U. vulgaris* transcriptome but not the *U. gibba* transcriptome. Despite these differences, the proportions of annotated GO categories were very similar (Figure 1).

The distribution of GO categories in *Utricularia* was also in line with previously published transcriptomic data from carnivorous species of *Sarracenia* [30]. This study used only a quarter of the data of the *U. vulgaris* transcriptome, without pooling various tissues or developmental stages. Despite methodological differences, the proportions of GO categories were very similar between *U. vulgaris* and *Sarracenia*. Only the categories “Response to stimulus” and “Biological regulation” were much higher in *Utricularia* than recorded for *Sarracenia*. This difference may reflect distinct life styles of both carnivorous genera. Whereas *Sarracenia* is a robust slowly growing terrestrial perennial plant, *Utricularia* is a fast growing aquatic plant which has to cope with sudden changes of environment (nutrient level, salinity, streaming or even temporary desiccation). Alternatively, the impact of very different data sets cannot be excluded. In this case, it would affect only the two GO categories, which appears unlikely.

### Examples of alternative splicing

Normalization of cDNA is recommended for the study of AS, because it increases the proportion of rare mRNAs, often represented by alternatively spliced transcripts. This approach revealed that 61.2% of intron-containing genes were alternatively spliced in *Arabidopsis* [27]. We cannot directly compare the extent of AS in *U. vulgaris* and *Arabidopsis*, because missing genomic information in *U. vulgaris* prevents the accurate assignment of splice variants. However, 23 largest isogroups in *U. vulgaris* (Additional file 2) matched *Arabidopsis* homologs with more than 3 splice variants belonging to 25% of the genes with the highest level of AS in *Arabidopsis* [27], which suggests that a similar set of genes is highly alternatively spliced in *U. vulgaris* and *Arabidopsis*.

The gene encoding ATP dependent RNA helicase (isogroup 00008) was shown to be alternatively spliced not only in *U. vulgaris*, but also in *U. gibba*. It participates in the control of mRNA splicing and export in *Arabidopsis* [25,26] and its expression is regulated by AS in this plant. It is therefore possible that the paralogs encoding ATP dependent RNA helicase (isogroup 00008) play similar roles in *Utricularia*.

We also documented extreme AS in the isogroup 00007 in *U. vulgaris* homologous to the gene for LCB-1-P lyase in *Arabidopsis*. The function of LCB-1-P lyase or sphingosine-1-phosphate (SPH-1-P) lyase in plants is not fully understood. Ng et al. [31], Coursol S et al. [32] described the role of SPH-1-P as a lipid messenger in guard cell abscisic acid (ABA) response. Nishikawa et al. [33] showed that LCB-1-P was degraded by LCB-1-P lyase (encoded by *AtDPL1*, At1g27980), which was located in the endoplasmic reticulum. LCB-1-P lyase regulates LCB-1-P content and through this activity participates in stomata closure and dehydration stress response in *Arabidopsis* [33,34].

Our *U. vulgaris* transcriptome was generated from submersed plant organs which did not develop stomata. However, we have verified that above-water flower stems of *U. vulgaris* contain stomata (Adamec, unpublished results). It is therefore possible that transcripts with retained introns represent a pool from which functional transcripts may be readily formed by additional splicing when LCB-1-P lyase becomes necessary. This protein may be needed when the submersed plant body continues to grow above water. A similar regulatory role of intron retention was observed, for example, in the fern *Marsilea vestita*, where transcripts with retained introns were stored in spores and spliced after germination [35].

The expression of the gene for LCB-1-P lyase in submersed plant organs lacking stomata may also suggest that it fulfills a distinct function in *Utricularia*, not associated with stomata. As SPH-1-P affects ion channels in guard cell protoplasts [32], we speculate that this lipid messenger may have a role in water pumping regulation in *Utricularia* traps, which is associated with potassium channels in trap bifid glands [36]. Finally, it is possible that extreme AS in the isogroup 00007 coding for LCB-1-P lyase does not have any regulatory function and represents an error in a complex splicing process. To our knowledge, AS of transcripts encoding LCB-1-P lyase has not been studied in any plant species. We cannot determine whether this also occurs in *U. gibba*, because non-normalized transcriptomes have only limited potential to detect AS.

### Duplication of the *EIN2* gene in *U. vulgaris*

We found a duplication of *EIN2* gene in *U. vulgaris* transcriptome. This gene is essential for ethylene signaling and occurs in a single copy in many plant species and its duplication is rare among angiosperms [37,38]. Two *EIN2* paralogs undergoing accelerated evolution were recently identified in *Lotus japonicus* [39]. They regulate not only a response to ethylene, but also nodulation in the course of symbiosis with rhizobia. The two *EIN2* paralogs in *Utricularia vulgaris* may be important for the interaction between plants and microbes, similar to the role of the two *EIN2* genes in the symbiosis between leguminous plants and bacteria [39]. We speculate that the duplication of the *EIN2* gene occurred early in the evolution of the genus *Utricularia* and might have been associated with the transition to a carnivorous life-style. Subsequently, one copy was lost in *U. gibba*. Similar Ka/Ks ratios in pairwise comparisons of *Utricularia EIN2* genes do not indicate any shift in function. However, it should be emphasized, that only parts of the *U. vulgaris EIN2* genes, confirmed by Sanger sequencing, were analyzed. The examination of additional species of Lentibulariaceae regarding *EIN2* multiplication will shed light on the evolution and function of this important gene.

### Low sequence similarity between putative *U. gibba*-*U. vulgaris* orthologs

The median value of sequence similarity among 12,267 putative orthologous pairs (measured as high-scoring segment pair of BLAST alignments) between the two *Utricularia* species was only 87% (Figure 5), much lower than for example a median ortholog similarity between two *Corylus* species (98%)- [40] or between chimpanzee and human (about 93.7%- [41]) which belong to different genera. *U. vulgaris* and *U. gibba* are classified in the same generic section *Utricularia* [1,5], although they are not sister species. The reason for the high divergence between *U. gibba* and *U. vulgaris* appears to be a high substitution rate, described in Lentibulariaceae as one of the characteristics of the plant carnivorous syndrome [11,42]. However, these two species still displayed very high sequence similarity in orthologs encoding ribosomal proteins, components of respiratory chain and cytoskeletal proteins, which were ultraconserved across the plant kingdom. We also observed a high conservation of some genes involved in root-associated function, which were generally less conserved among angiosperms. This could be explained by a functional shift shared by the two rootless aquatic *Utricularia* species.

### The loss of root-associated genes

We found a perfect coincidence between the absence of root-associated genes in the *U. gibba* genome [13] and the absence of their counterparts in the *U. vulgaris* transcriptome. The correspondence between both *Utricularia* species was also observed in additional root-associated genes, not specifically analyzed by [13] (Additional file 6). Although the absence of particular genes in our transcriptome may be due to intrinsic incompleteness of any experimental transcriptomic data, the high coincidence between genomic and transcriptomic gene occurrences in two species suggests that many root-associated genes are indeed missing in the *U. vulgaris* genome. It is probable that the loss of root-associated genes had occurred already in the ancestor of *U. gibba* and *U. vulgaris*. The comparison of the presence or absence of root-associated genes in additional *Utricularia* species will be very useful for understanding the adaptation to an aquatic rootless carnivorous life-style.

Besides gene losses, gene duplications could be also very informative regarding the evolution and consequences of aquatic carnivory in plants. For example, the duplication of *ARF16* in *U. gibba* [13] was also observed in the *U. vulgaris* transcriptome. In contrast, the *EIN2* duplication event was unique for *U. vulgaris*.

## Conclusions

Our study is the first example of comparative transcriptomics in the species-rich genus *Utricularia*. We compared the transcriptome of *U. vulgaris* with the previously published transcriptome of *U. gibba* [12] and confirmed a general similarity of their expression profiles. Both *Utricularia* species displayed some distinctions from the *Sarracenia* transcriptome [30] due to the aquatic life-style different from a terrestrial carnivory. Average sequence similarity of the putative orthologs between *U. vulgaris* and *U. gibba* was lower than 90%, which could be caused by elevated substitution rate in this genus [11]. A strong correspondence in presence or absence of root-associated genes between *U. vulgaris* and *U. gibba* suggests that the loss of some root-specific genes had occurred before the two rootless species separated. Future transcriptome and genome comparisons over numerous *Utricularia* species will certainly deepen our understanding of plant evolution and adaptation.

## Methods

### Plant material

We utilized *U. vulgaris* from three sources for RNA extraction: (1) sterile plants from a meristem tissue culture (courtesy of Ing. Kamil Pásek, Ostrava-Poruba, Czech Republic), growing in a half-strength Gamborg B5 liquid medium with 500 mg l^−1^ KNO_3_, microelements, vitamins, and 2.5% sucrose, but without other organic substances [43]; (2) sterile plants transplanted axenically from tissue culture into an aerated mineral medium [44], with reduced amounts of phosphorus and nitrogen where plants were grown in 16/8 h light/dark regime of fluorescent light at 23°C for 4 weeks, attaining the length of approximately 20 cm; and (3) plants with colonized traps, approximately 35 cm in length, cultivated in a 750 litre outdoor plastic container closely simulating natural dystrophic conditions [45], with the litter of robust sedges (*Carex* spp.) used as a substrate [45] and partial shading (usually 20-50% of incident irradiance). Plants cultivated outdoor were occasionally fed fine zooplankton prey to support growth. No experiments on humans or animals, or cloning experiments were carried out. All activity complied with ethics rules.

Tissue for RNA extraction was harvested in the middle of the dark period to reduce the amount of Rubisco-coding mRNA in samples, frozen immediately in liquid nitrogen, and stored at-70°C until further processing.

### RNA extraction, cDNA library construction, and 454 sequencing

Total RNA was extracted from entire shoots, including leaves and traps, from all three sources described above. Inflorescences were not used in this study. Approximately 800 mg of fresh weight (2 individuals) of whole shoots in three replicates from each source were ground in liquid nitrogen using a mortar and pestle and RNA was extracted using RNeasy Plant Midi Kit (Qiagen Inc., Valencia, CA, USA). The results of the parallel extractions were pooled in equal proportions into a single sample and the quality and quantity of the extracted RNA were verified by gel electrophoresis and by spectrophotometer, measuring the 230/260 ratio with Biophotometer (Eppendorf, Germany).

A pooled RNA sample was provided to GATC Biotech (Konstanz, Germany) for the construction of a cDNA library and subsequent sequencing. Library construction involved DNAselection for polyadenylated (polyA+) transcripts to enrich for protein-coding mRNAs, DNase I treatment and normalization through denaturation/reassociation of cDNA, to improve the representation of low-copy transcripts and thereby maximize gene discovery. The resulting library was sequenced with a full picotiter plate on a 454 GS-FLX sequencer with Titanium reagents (Roche Applied Science, Indianapolis, IN, USA), using standard 454 protocols.

### Transcriptome assembly and annotation

Raw reads obtained by 454 pyrosequencing were submitted to European Nucleotide Archive under the study accession number PRJEB8057 (http://www.ebi.ac.uk/ena/data/view/PRJEB8057).

We assembled our raw reads with Newbler 2.7 using the following command line settings:–cdna,–it 500,–ig 1000,–icc 200. The subsequent annotation steps excluded singletons that were <100 bp in length. Annotation of isotigs (contigs assembled by Newbler and roughly corresponding to individual transcripts) and singletons (transcripts represented by single reads) was done using the BLAST2GO pipeline [46] with the following parameters: Annotation rule cutoff 55, e-value 10^−6^, Hit-HSP overlap 0, GO weight 5.

MEGAN v 5.7.0 [47] with default parameters was used to get the taxonomical annotation of isotigs and singletons. MEGAN uses BLASTX output and LCA (Lowest Common Ancestor) classifier based on the actual NCBI taxonomy to obtain the taxonomical annotations.

### Search for orthologs

The orthologous transcripts between *U. vulgaris* and *U. gibba* were identified by means of reciprocal BLAST search [48]. We used the transcriptome of *U. vulgaris* containing both isotigs and the singletons which passed quality filtering (min phred score 25, min length 100, max ambiguities 0, max homopolymer 8). As the *U. gibba* transcriptome published by Ibarra-Laclette et al. [12] might have been incomplete, we downloaded the database 19475-mRNA, constructed from the complete genome of *U. gibba*, from CoGe website (https://genomevolution.org/CoGe/-id36222). We generated local databases and used BLASTN, threshold e–10, to retrieve the best hits in reciprocal BLAST searches. Reciprocal BLAST outputs were compared by RBH (reciprocal blast hit) script and putative orthologs which appeared as the same pair in both BLAST outputs were identified. The similarity of orthologs in % was based on high-scoring segment pair (HSP) of BLAST alignments.

The orthologs between *U.vulgaris* and *Arabidopsis* (TAIR10_prot_20101214) were searched in a similar way, with BLASTX (threshold e–10) and TBLASTN (threshold e–10) instead of BLASTN, which means that predicted protein sequences were aligned.

### The analysis of large isogroups

We selected the isogroups containing more than 45 isotigs in *U. vulgaris* and more than 10 isotigs in *U. gibba* and found their *Arabidopsis* counterparts using the BLASTX search in TAIR (www.arabidopsis.org). We assigned their functions based on TAIR annotations. We aligned all isotigs from two selected isogroups of *U. vulgaris*, each displaying an extremely high number of isotigs, by MUSCLE implemented in Geneious 7.1.5. We identified introns retained in the transcripts with the help of Layout output from Newbler 2.7 and by comparison with gene structures in *Arabidopsis* (TAIR).

### GO enrichment analysis

We performed the analysis of enrichment of particular GO categories among putative *U. gibba*–*U. vulgaris* orthologs by means of AgriGO (http://bioinfo.cau.edu.cn/agriGO) [29]. We input the list of annotated putative orthologs as a *customized annotated reference* and the list of orthologs with specific similarity as *a customized annotation*. We applied Yekutieli multi-test adjustment, significance level 0.05 and a minimum number of mapping entries of 4.

### RT PCR confirmation

To verify transcript sequences inferred from the 454 assembly, we collected *U. vulgaris* plants grown outdoor and isolated total RNA by RNeasy Plant Mini Kit (Qiagen). RNA was treated with DNase I (DNA-free, Ambion, TX, USA) to eliminate a DNA contamination. The treatment was repeated until no amplification was observed in PCR reaction with RNA as a template. One μg of RNA and oligo dT primers (500 ng) were heated 5 min at 65°C, chilled on ice and mixed with Transcriptor buffer (Roche Scientific, Mannheim, Germany), 0.5 μl of Protector RNase Inhibitor (Roche Scientific), 2 μl of 10 mM dNTPs and 10 units of Transcriptor Reverse Transcriptase (Roche Scientific). The first strand of cDNA was synthesized at 55°C for 30 min and then amplified with the primers (Additional file 4) targeted to isotig 00405 (isogroup 00007). PCR was run in a T Gradient cycler (Biometra, Goettingen, Germany) with Taq polymerase (Promega, Madison, WI, USA) under the following conditions: initial denaturation 2 min at 94°C; 36 cycles consisting of 40 s at 93°C, 45 s at given annealing temperature and 2 min at 72°C; final extension 10 min at 72°C.

### PCR confirmation

Genomic DNA from *U. vulgaris* plants grown outdoor was isolated by the sorbitol extraction [49]. PCR with the primers (Additional file 4) targeted to isotigs 00304 or 00306 (isogroup 00006) was run as described above at annealing temperature 60°C. PCR products were Sanger sequenced and partial genomic sequences corresponding to two paralogs of *EIN2* in *U. vulgaris* were deposited in GenBank under the numbers KP279653 and KP279654.

### Phylogenetic analyses

We downloaded *EIN2* sequences of *U. gibba* (Sc00341_g15851 from CoGe-id36222), *Genlisea aurea* (EPS70151), *Solanum tuberosum* (XM006354273), *Petunia hybrida* (AY353249), *Populus trichocarpa* (XM002326149*), Lotus japonicus* (chr5.CM1729.230 and chr1. CM0012.1100), *Dianthus caryophyllus* (HQ441183), *Arabidopsis thaliana* (NM12040*), Zea mays* (AY359584), and *Sorghum bicolor* (XM002457067). We aligned these angiosperm EIN2 sequences with partial protein sequences EIN2-1 and EIN2-2 from *U. vulgaris* and created a 1,361 amino acid long gapped-alignment in Geneious 7.1.5. We then adopted a maximum parsimony (MP) approach with stepwise addition to the starting tree, no topology constraints, tree bisection-reconnection (TBR) swap, and 1000 bootstrap pseudoreplicates to construct a 60% consensus tree in PAUP*4 v10 [50]. Gaps were treated as missing characters. To confirm phylogenetic positions of the *EIN2* paralogs in *Utricularia* we performed Maximum Likelihood (ML) analysis in PAUP*4 v10 [50] of the same nucleotide alignment under HKY85 evolution model, using the same tree construction methods as described above, 1000 bootstrap pseudoreplicates.

### Availability of supporting data

We summarized the results of our *U. vulgaris* transcriptome assembly and annotation and created a web-accessible database (http://utricularia.prf.jcu.cz/index.php) which can be easily searched by BLAST or annotation. Raw reads obtained by 454 pyrosequencing were submitted to European Nucleotide Archive under the study accession number PRJEB8057 (http://www.ebi.ac.uk/ena/data/view/PRJEB8057). The sequences of *EIN2* paralogs were deposited in GenBank under the numbers KP279653 and KP279654. The data sets undelying phylogenetic *EIN2* studies was submitted to TreeBase under the ID 17158. The sequences of *U. gibba* were obtained from the study of Ibarra-Laclette et al. [12] (doi:10.1186/1471-2229-11-101).

## Additional files

Additional file 1:
**Dendogram showing number of MEGAN assigned** ***U. vulgaris*** **(A) and** ***U. gibba*** **(B) singletons.**
Additional file 2:
**Isogroups with numerous isotigs in** ***U. vulgaris*** **and** ***U. gibba.***
Additional file 3:
**Layout of the isotigs comprising the isogroup 00007.** Exons are marked in yellow, contigs corresponding to exons and introns are blue. Additional file 4:
**PCR primers used in this study.**
Additional file 5:
**GO categories significantly enriched among** ***U. gibba*** **–** ***U. vulgaris*** **putative orthologs with sequence similarity higher than 93%.**
Additional file 6:
**Root-associated genes in** ***U. vulgaris*** **and** ***U. gibba.***

## Abbreviations

*ARF*: *AUXIN RESPONSE FACTOR*
AS: Alternative splicing
*BRN*: *BEARSKIN*
DDBJ: DNA Data Bank of Japan
*EIN*: *ETHYLENE INSENSITIVE* GO, Gene Onthology
HSP: high scoring segment pair
*IAA*: *INDOLE-3-ACETIC ACID INDUCIBLE*
LCB-1-P: long-chain base 1-phosphate
MP: maximum parsimony
RBH: reciprocal blast hit
*RHD*: *ROOT HAIR DEFECTIVE*
TBR: tree bisection-reconnection
*SBM*: *SOMBRERO*
SPH-1-P: sphingosine-1-phosphate
*TIP*: *TIP GROWTH DEFECTIVE*
*WER*: *WEREWOLF*
