# Supplementary figures and images for: The transcriptome of Utricularia vulgaris, a rootless plant with minimalist genome, reveals extreme alternative splicing and only moderate sequence similarity with Utricularia gibba
Source: BMC Plant Biol. 2015 Mar 7;15:78. doi: 10.1186/s12870-015-0467-8 (PMC4358910; doi:10.1186/s12870-015-0467-8)

A.

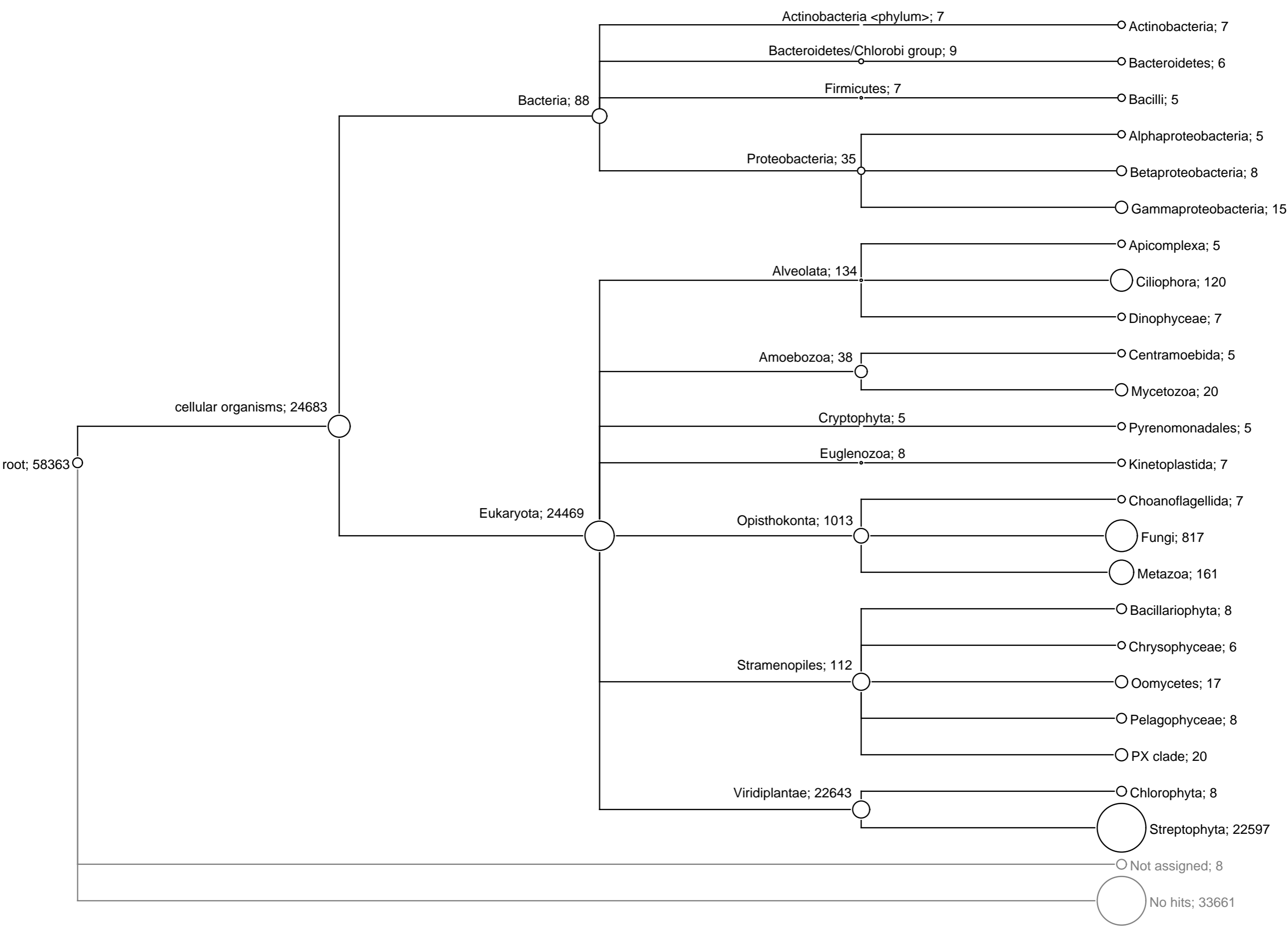

B.

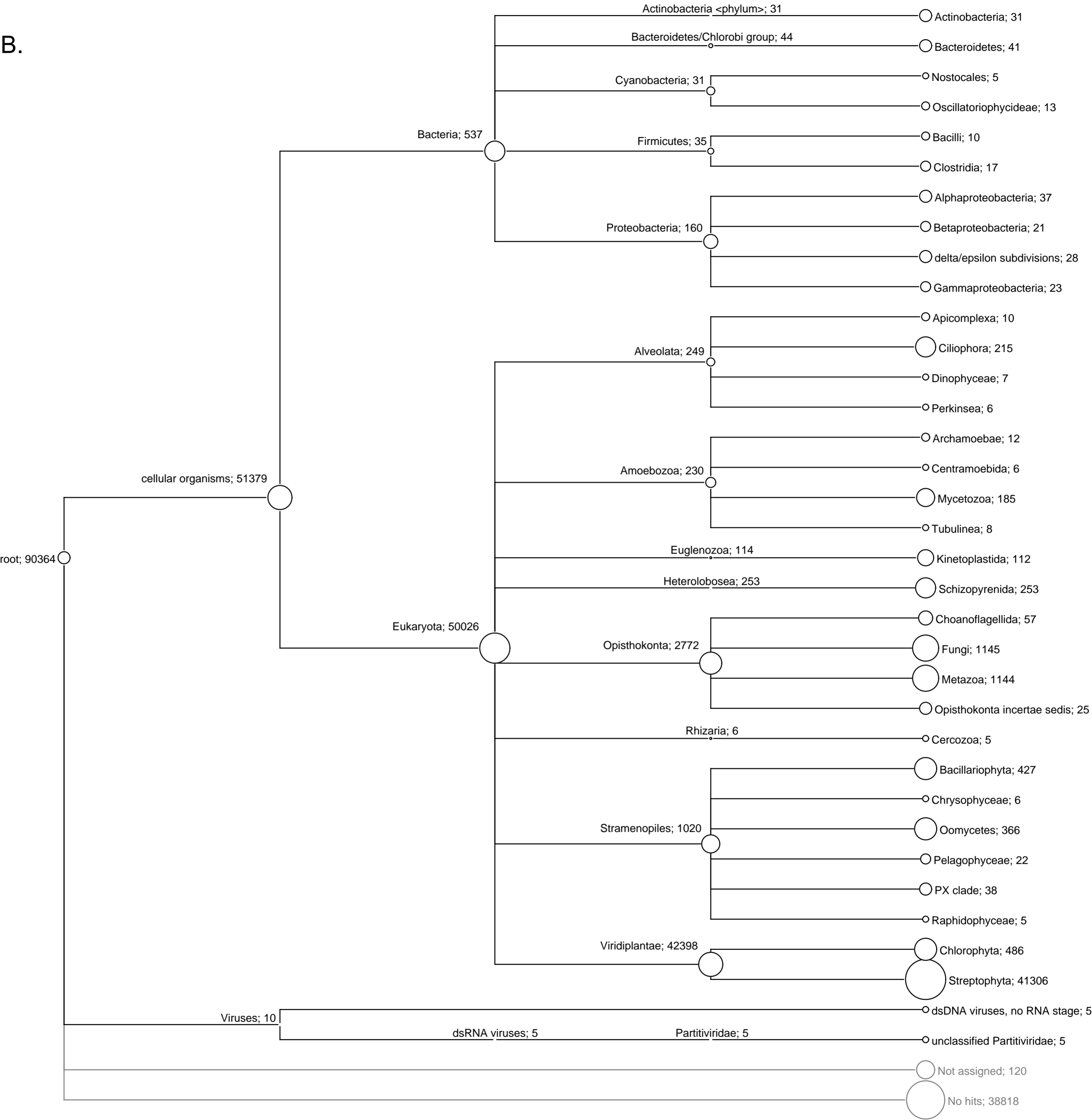

Supplement: Additional file 1: — Dendogram showing number of MEGAN assigned U. vulgaris (A) and U. gibba (B) singletons. [file 12870_2015_467_MOESM1_ESM.pdf]
