# Supplementary material for: The transcriptome of Utricularia vulgaris, a rootless plant with minimalist genome, reveals extreme alternative splicing and only moderate sequence similarity with Utricularia gibba
Source: BMC Plant Biol. 2015 Mar 7;15:78. doi: 10.1186/s12870-015-0467-8 (PMC4358910; doi:10.1186/s12870-015-0467-8)
Supplement: Additional file 4: — PCR primers used in this study. [file 12870_2015_467_MOESM4_ESM.doc]

**Table S1.** PCR primers used in this study

**Isogroup 00006**

**UV304_F1**: tgagttctgtgaggtgccatt

**UV304_R1**: tgacataacttgctcgtgtgc

**UV306_F1**: aaacctgtgacgtgccattt

**UV306_R1**: atctgcaaagaggcagcaat

**Isogroup 00007**

**UV405_F1** ATTTGAAGCCGAAGTTGTGG

**UV405_R1** AACATGGAGCTGGCGATTAC

**UV405_F2** CCTGGATGTCCTGTTTCTCC
